# Rate of Mortality among the Students of the Medical Institute of Louisville

**Published:** 1845-02

**Authors:** 


### RATE OF MORTALITY AMONG THE STUDENTS OF THE MEDICAL INSTITUTE OF LOUISVILLE.

In its eight sessions the Institute has had 1594 pupils. For our present purpose we shall call it 1600, or 200 annually. Many of them arrive in October, some as early as the latter part of September, and the candidates for graduation, with a number of others, remain till the 10th or 12th of March. Some continue throughout the vacation. Thus, although all are not in by the first of November, and a very considerable number depart before the end of February, still it cannot be far from the truth to regard the whole 200 as sojourning in Louisville for one-third of the year. Three sessions, then, would give a residence equal to a year to 200, and eight sessions would give a residence, for the same number, equal to two years and eight months; but as one month of the present session has yet to run, we shall throw off two months, and come to the conclusion that the residence of medical students in Louisville, since the foundation of the school in 1837, has been equal to that of 200 for two years and a half; or 500 for one year. Now, of the whole but one has died during the sessions of the school. Several, it is true, have left it in bad health, and *may* have died after reaching home, and before the expiration of the session, but no case of that kind is known to the Faculty.

Taking the average annual date of mortality, in the community at large, at the low number of one in fifty, that in the Institute being only one in 500, is but a tenth part as great. We confess that this result surprises us. To what shall we ascribe it? In casting about for the causes, the following occur to us:

1. A large number of the deaths, in general society, are of children, and aged persons.

2. A great number are from the fevers of summer and early autumn.

3. Students, during the session, are but little exposed to inclemencies of weather.

4. With a rare exception, they do not drink ardent spirits, or wine, or malt liquors, to an extent injurious to health, and a majority either practice total abstinance, or taste intoxicating drinks but moderately, and at long intervals.

5. Students of medicine, during their attendance on the lectures, make *early* application to the professors for medical aid, and generally submit to the required treatment, whereby many of their diseases are arrested in the beginning.

6. Louisville is not far enough north to be subject to the typhoid fevers, which prevail in higher latitudes in winter; and even its pulmonary inflammations are neither frequent nor violent.

Of the pupils of the Institute, 783 or within a small fraction of one-half, have been from the States south of the northern boundary of Tennessee, in latitude 36° 30′. The single death which has occurred is one of them, a native of North Carolina, but a resident of Mississippi. Thus there has been only one death out of 250 southern young men residing in Louisville for a year, the whole being in cold weather. This fact proves conclusively that this city is within those limits, to which the young men of the south may with safety be sent to pass the winter. Indeed, we are disposed to believe, from observation, as well as theory, that many of them are benefitted by a winter sojourn here, as it respects the ravages which their constitutions have experienced from the protracted heat and malarious atmosphere of the south. It is an admitted fact, that pneumonia prevails in the south and often proves fatal, because, the enfeebled constitutions of the people, will not endure an active antiphlogistic treatment. The lungs are destroyed by engorgement, unaccompanied by a well developed inflammatory diathesis. But after an individual, enfeebled during summer and autumn, has spent a few weeks in this higher latitude, his vital powers are so elevated, that true inflammation, and a genuine phlogistic diathesis are set up, and yield to free depletion and the ordinary antiphlogistic medicines. To generalize, we venture to express the opinion that pneumonia is more manageable on the banks of the Ohio than the Alabama river—on the Upper than the Lower Mississippi. D.

